# Predictive Value of a Gastric Microbiota Dysbiosis Test for Stratifying Cancer Risk in Atrophic Gastritis Patients

**DOI:** 10.3390/nu17010142

**Published:** 2024-12-31

**Authors:** Alice Zaramella, Diletta Arcidiacono, Miriam Duci, Clara Benna, Salvatore Pucciarelli, Alberto Fantin, Antonio Rosato, Valli De Re, Renato Cannizzaro, Matteo Fassan, Stefano Realdon

**Affiliations:** 1 Department of Surgery, Oncology and Gastroenterology (DiSCOG), University of Padua, Via Giustiniani 2, 35128 Padua, Italy; alice.zaramella@unipd.it (A.Z.); clara.benna@unipd.it (C.B.); puc@unipd.it (S.P.); antonio.rosato@iov.veneto.it (A.R.); 2 Gastroenterology Unit, Veneto Institute of Oncology IOV-IRCCS, Via Gattamelata 64, 35128 Padua, Italy; diletta.arcidiacono@iov.veneto.it (D.A.); alberto.fantin@iov.veneto.it (A.F.); 3 Department of Women’s and Children’s Health, University of Padova, 35128 Padova, Italy; ducimiriam@gmail.com; 4 Pediatric Surgery Unit, Division of Women’s and Children’s Health, Padova University Hospital, 35128 Padova, Italy; 5 UOC Immunology and Molecular Oncology, Veneto Institute of Oncology IOV-IRCCS, Via Gattamelata 64, 35128 Padua, Italy; 6 Immunopathology and Cancer Biomarkers, Centro di Riferimento Oncologico di Aviano, National Cancer Institute, IRCCS, 33081 Aviano, Italy; vdere@cro.it; 7 Oncological Gastroenterology, Centro di Riferimento Oncologico di Aviano (CRO), National Cancer Institute, IRCCS, 33081 Aviano, Italy; rcannizzaro@cro.it; 8 Department of Medical, Surgical and Health Sciences, University of Trieste, 34127 Trieste, Italy; 9 Veneto Institute of Oncology IOV-IRCCS, Via Gattamelata 64, 35128 Padua, Italy; matteo.fassan@unipd.it; 10 Surgical Pathology Unit, Department of Medicine (DIMED), University of Padua, Via Gabelli 61, 35121 Padua, Italy

**Keywords:** microbiome, gastric cancer, *Helicobacter pylori*

## Abstract

Background/Objectives: Gastric cancer (GC) incidence remains high worldwide, and the survival rate is poor. GC develops from atrophic gastritis (AG), associated with *Helicobacter pylori* (*Hp*) infection, passing through intestinal metaplasia and dysplasia steps. Since *Hp* eradication does not exclude GC development, further investigations are needed. New data suggest the possible role of unexplored gastric microbiota beyond *Hp* in the progression from AG to GC. Aimed to develop a score that could be used in clinical practice to stratify GC progression risk, here was investigate gastric microbiota in AG *Hp*-negative patients with or without high-grade dysplasia (HGD) or GC. Methods: Consecutive patients undergoing upper endoscopy within an endoscopic follow-up for AG were considered. The antrum and corpus biopsies were used to assess the microbiota composition along the disease progression by sequencing the 16S ribosomal RNA gene. Statistical differences between HGD/GC and AG patients were included in a multivariate analysis. Results: HGD/GC patients had a higher percentage of *Bacillus* in the antrum and a low abundance of Rhizobiales, Weeksellaceae and *Veillonella* in the corpus. These data were used to calculate a multiparametric score (Resident Gastric Microbiota Dysbiosis Test, RGM-DT) to predict the risk of progression toward HGD/GC. The performance of RGM-DT in discriminating patients with HGD/GC showed a specificity of 88.9%. Conclusions: The microbiome-based risk prediction model for GC could clarify the role of gastric microbiota as a cancer risk biomarker to be used in clinical practice. The proposed test might be used to personalize follow-up program thanks to a better cancer risk stratification.

## 1. Introduction

Gastric cancer (GC) in 2022 shows 968,000 new cases and about 660,000 deaths, ranking the disease as fifth in terms of both incidence and mortality worldwide [1]. In Italy, in 2022, about 14,700 new diagnoses were estimated (8800 for men and 5900 for women), and the 5-year survival rate is 30% in males and 35% in females. GCs are associated with environmental factors such as diet, lifestyle, and infectious agents [2,3]. Most GCs are adenocarcinoma and develop through a cascade of precancerous lesions (Correa’s cascade) starting with atrophic gastritis (AG) and passing through intestinal metaplasia, low-grade and high-grade dysplasia (HGD), and finally GC [4]. The extent and the severity of gastric atrophy (i.e., stages III and IV according to the Operative Link for Gastritis Assessment [OLGA] staging system) are also associated with the risk of developing GC [5].

Moreover, due to its role in the inflammation of gastric mucosa and AG onset, long-standing *Helicobacter pylori* (*Hp*) infection is widely recognized as the main risk factor for GC development [1,6,7]. On the one hand, the presence of *Hp* causes a hypochlorhydria state that provides a more favorable environment for the colonization of other bacteria, while on the other hand, *Hp* presence is also correlated with a reduction in bacterial diversity and richness [8]. Indeed, several studies have demonstrated that a distinctive flora colonizes the stomach and is significantly different from oral and esophageal microbiota, including bacteria and fungi [9,10,11]. Considering that less than 3% of chronic *Hp*-infected patients develop GC [12] and that eradicating *Hp* can restore gastric diversity but does not completely reduce chronic inflammation [13,14], other factors may be involved in GC development. The gastrointestinal microbiome could influence the immune system, inflammation, and metabolism [15,16]. The so-called “dysbiosis condition,” representing the equilibrium perturbation in terms of abundance and composition in the microbiome, has been correlated to different gastrointestinal diseases, including cancer [17]. Many studies focused on a better comprehension of resident bacterial flora during gastric diseases [18,19,20,21,22,23], but data comparison between different studies is challenging.

This study aimed to explore the resident gastric microbiota in *Hp*-negative AG patients without dysplasia or GC and in AG patients with HGD or GC. The main goals were to identify bacteria possibly involved in gastric carcinogenesis and to develop a “dysbiosis test” potentially able to stratify cancer risk in AG patients that could be used in clinical practice.

## 2. Materials and Methods

### 2.1. Study Design and Patients Included in the Study

Consecutive patients undergoing a follow-up upper endoscopy at IOV-IRCCS Digestive Endoscopy Unit between 10 January 2014 and 10 January 2017 because of an already-known diagnosis of AG were considered. The exclusion criteria were the following:

(i) Presence of other types of cancer;
(ii) Presence of autoimmune atrophic gastritis;
(iii) Taking antibiotics/probiotics/Proton Pump Inhibitors (PPIs) within 3 months;
(iv) Currently positive for *Hp* infection;
(v) Previous gastric/esophageal surgery.

Positivity to *Hp* was tested by the pathologist who performed the modified Giemsa staining of the biopsy specimens. Only *Hp*-negative patients with a final diagnosis of mild AG (OLGA stages I–II [5]), patients with severe AG (OLGA stages III–IV [5]) without dysplasia or cancer, and patients with severe AG and HGD or GC (resulting in T1N0 after complete staging) were included. All pathological diagnoses were confirmed by two pathologists who analyzed the same biopsy specimen. All patients provided written informed consent according to the Declaration of Helsinki. Informed consent was obtained from all the patients, and all the information was recorded anonymously according to the regulations of our institution.

**Figure 1:**
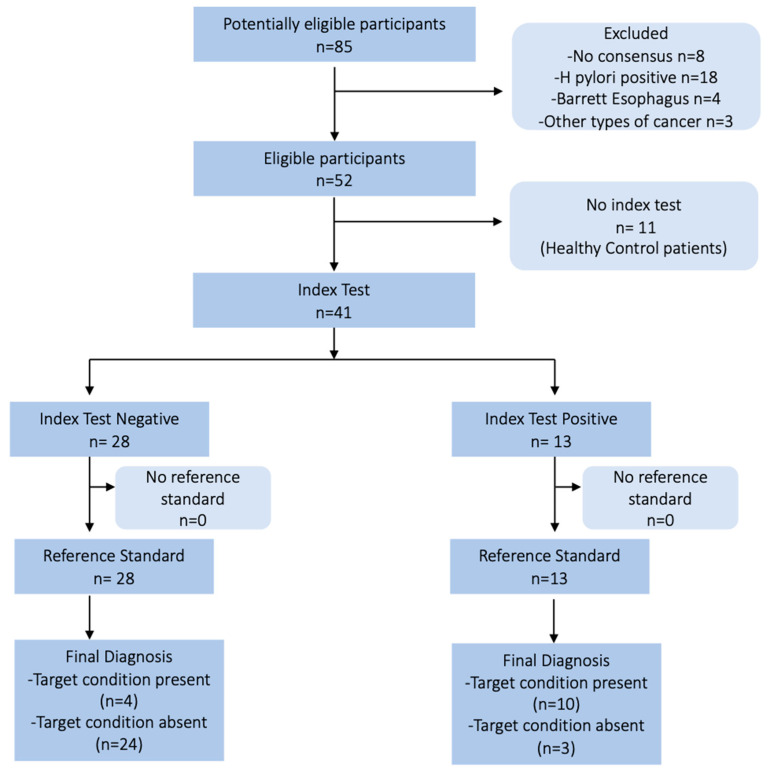
The schematic flow chart of the study design and the patients’ course.

Healthy *Hp*-negative subjects with normal gastric mucosa undergoing upper endoscopy during the same period were considered control patients. A flow diagram of patients included in the study is reported in Figure 1. During endoscopy, biopsies from the antrum and corpus were collected, fixed in formalin, and sent to the Pathology Unit for histological examination. Information on the gastric microbial profile was obtained from additional biopsies collected from the antrum and corpus and immediately frozen in liquid nitrogen. Biopsy specimens were stored at −80°C in the University of Padua—Surgical Unit 3 Biobank. Data from follow-up upper endoscopies were recorded until 10 January 2023 to evaluate the negative predictive value of our test.

### 2.2. DNA Extraction and Sequencing Analyses

Frozen biopsies collected from all patients during endoscopy were used to evaluate the microbial composition directly on the gastric samples. The bacterial DNA extraction and sequencing protocols were the same as those used in a previous study [24]. In brief, biopsies were mechanically destroyed first using Tissue Lyser II (Qiagen, Hilden, Germany). Bacterial DNA was extracted with the QIAamp DNA Microbiome Kit (Qiagen, Hilden, Germany). Purified bacterial DNA was amplified by targeting the V3–V4 region of the bacterial 16S rRNA gene. Libraries were prepared using the QIAseq 16S region panel for the V3–V4 region (Qiagen, Hilden, Germany). Library quantification was evaluated by real-time PCR using the QIAseq Library Quant (Qiagen, Hilden, Germany). Through the Agilent TapeStation (Santa Clara, CA, USA), the quality of libraries was assessed. Amplicons were sequenced at the end using the Illumina Miseq platform (Miseq Reagent Kit v3 600 cycles, Illumina, San Diego, CA, USA).

The 16S rRNA raw sequences were merged, demultiplexed, trimmed down to 250 nucleotides, and filtered. High-quality filtered reads were clustered into operational taxonomic units (OTUs) using CLC Genomics Workbench and CLC Microbial Genomics Module v.21 (Qiagen, Hilden, Germany). Taxonomic assignment of sequences was carried out based on the SILVA (v132) database with 97% similarity. Clusters of OTUs composed of only one read were discarded. OTUs belonging to Eukarya, Archea, chloroplast, cyanobacteria, and mitochondria were removed from further downstream analysis.

Inter-sample diversity (beta diversity) was calculated using Bray–Curtis, Jaccard, unweighted and weighted UniFrac matrices and visualized as a principal coordinate analysis plot (PCoA). Moreover, sample biodiversity (alpha-diversity) was estimated according to different microbial metrics such as the Shannon and Chao-1 indices, and Faith’s phylogenetic distance (FD).

### 2.3. Statistical Analysis

The one-way ANOVA analysis of variance or the Kruskal–Wallis test (KW) was used for multiple-group comparisons. Cuzick’s trend test was performed to measure the trend of microbial relative abundance along the disease progression. The Mann–Whitney U test (MW) was used to assess differences between pre-cancerous patients and patients who progressed through cancer. For correlation analysis, Spearman’s rank correlation test was performed. A univariate and multivariate logistic analysis was performed to estimate the association between microbiota features and the disease state, following a previous study [24]. Briefly, the microbial characteristics that turned out to be significantly different between patients with pre-neoplastic lesions and patients who progressed through dysplasia or cancer were included in an univariable logistic analysis. Those with a *p* < 0.05 were further included in the multivariate logistic analysis to determine the risk factors for cancer progression in pre-cancerous patients. The model’s accuracy was finally tested with the receiver operating characteristic (ROC). A *p*-value lower than 0.05 was assumed to indicate a significant difference. Data analyses were performed using STATA 12.0 (StataCorp. 2011. Stata Statistical Software v.16: Release 12. College Station, TX: StataCorp LP, USA) and GraphPad Prism9 (GraphPad Software Inc., La Jolla, CA, USA). Alpha diversity measurements were analyzed by one-way analysis of variance (ANOVA) with the false discovery rate (FDR) correction. The Permutational Multivariate Analysis of Variance (PERMANOVA) was used for the multi-group comparison of beta diversity.

## 3. Results

### 3.1. Characteristics of the Population and Quality of Sequencing

According to histological findings, a total of 52 patients were included and divided into four groups: 11 healthy subjects (CTRL group), 13 patients with mild AG (mAG group), 14 patients with severe AG (sAG group), and 14 patients with severe AG and HGD or GC (HGD/GC group). The patient’s characteristics are summarized in Table 1.

**Figure 2:**
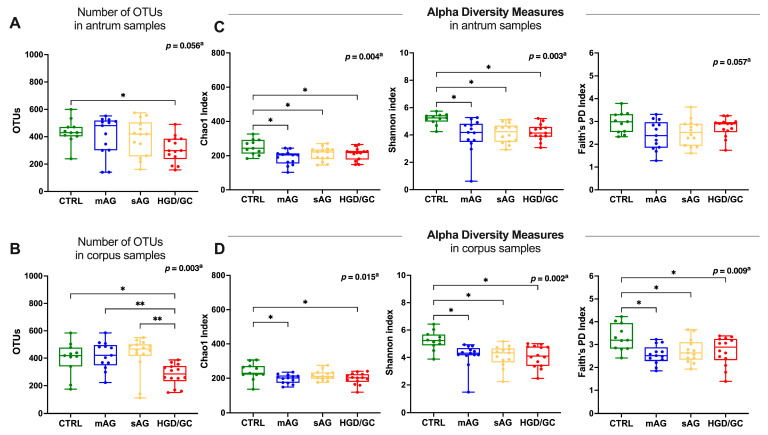
Quality of sequencing and alpha diversity measures. (**A**,**B**) Comparison of the number of OTUs among the patient groups in the (**A**) antrum and (**B**) corpus. (**C**,**D**) Comparison of alpha diversity measures in each group studied in the antrum (**C**) and corpus (**D**) biopsies. Statistical analyses were performed with one-way ANOVA. Post hoc analyses are annotated as * *p* < 0.05, ** *p* < 0.01. Box plots represent the median, interquartile range, and lower and minimum values. Each dot represents an individual patient. CTRL are healthy subjects; mAG: patients with mild atrophic gastritis (i.e., OLGA stages I–II); sAG: patients with severe atrophic gastritis (i.e., OLGA stages III–IV); HGD/GC: patients with severe AG and HGD or GC.

In the antrum, 31,400,304 high-quality reads were obtained, with an average of 603,852 reads per sample for the microbiota analysis. After filtration and removal of chimeric reads, 9,137,385 reads in OTUs were obtained. A total of 2179 OTUs were found. The number of OTUs in HGD/GC patients was significantly lower than in CTRL (post hoc result *p* = 0.010, Figure 2A).

In the corpus, 33,098,579 high-quality reads were obtained, with an average of 636,511 reads per sample. After filtration and removal of chimeric reads, 9,949,803 reads in OTUs were obtained. A total of 2427 OTUs were found. The number of OTUs in HGD/GC patients was significantly lower than in any other studied group (post hoc result CTRL *p* = 0.001; mAG *p* = 0.002; sAG *p* = 0.001. Figure 2B). No differences were observed comparing the number of OTUs between the antrum and corpus in each stage of the disease (Figure S1 Supplemental Materials). Alpha diversity was analyzed as a measure of within-sample diversity through Chao1, Shannon, and Faith’s phylogenetic diversity (PD) indices. The richness of the microbiota (Chao1 index) was higher in CTRL patients compared to the other groups, both in the antrum and the corpus, as shown in Figure 2C,D. Furthermore, the diversity of microbiota was significantly reduced in each group compared to CTRL patients in both in corpus samples (Shannon index post hoc result *p* = 0.002, *p* = 0.002, *p* = 0.002 for mAG, sAG, CTRL, respectively, Figure 2D) and antrum samples (post hoc result *p* = 0.002, *p* = 0.004, *p* = 0.004 for mAG, sAG, CTRL, respectively, Figure 2C). Faith’s PD was also significantly decreased in patients with mAG (post hoc result *p* = 0.004), sAG (post hoc result *p* = 0.012), and HGD/GC (post hoc result *p =* 0.012) exclusively in corpus biopsies (Figure 2D).

**Figure 3:**
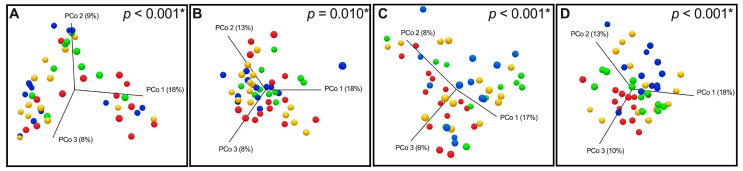
Principal coordinate analysis (PCoA) 3D plots of (**A**) weighted UniFrac in the antrum, (**B**) unweighted UniFrac in the antrum, (**C**) weighted UniFrac in the corpus, and (**D**) unweighted UniFrac in the corpus, in which samples are colored according to clinical outcome. Green dots represent CTRL patients, blue dots represent mAG patients, yellow dots represent sAG patients, and red dots represent HGD/GC patients. * PERMANOVA analysis.

To assess the diversity between groups, beta diversity was calculated using weighted and unweighted UniFrac phylogenetic distance matrices and shown on PCoA plots (Figure 3). The microbiota composition was significantly different in corpus samples between CTRL, mAG, sAG, and HGD/GC. Data describing the significance levels of PERMANOVA analysis of beta diversity are summarized in Table 2. Conversely, in antrum biopsies, significant differences were found between CTRL, sAG, and HGD/GC.

### 3.2. Microbiota Composition in the Antrum and Corpus Along the Disease

**Figure 4:**
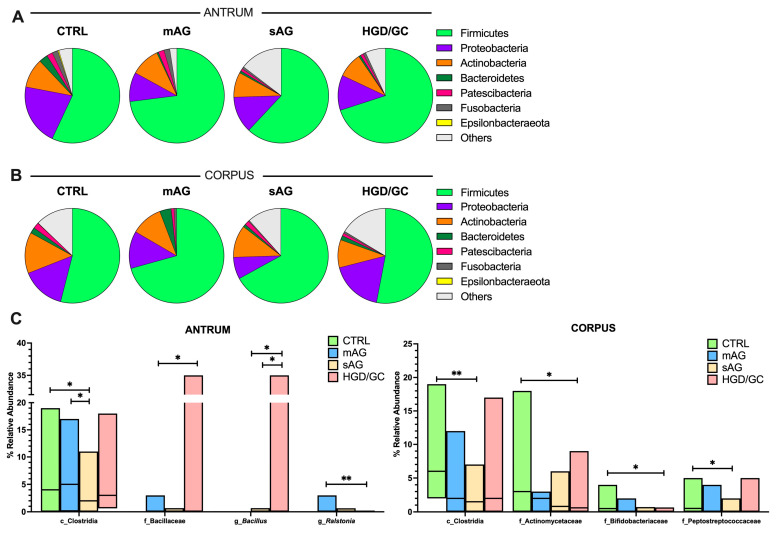
(**A**,**B**) Pie charts representing the relative abundance of the main phyla colonizing the gastric tissues in the antrum (**A**) and in the corpus (**B**) of all subjects and in each group of patients considered. Data are shown as the median values. Phyla with a relative abundance higher than 0.005% are plotted. (**C**) Bacteria significantly changed in gastric microbiota during GC development in the antrum and the corpus. Post hoc analyses are annotated as * *p* < 0.05, ** *p* < 0.01. CTRL: healthy subjects; mAG: patients with mild atrophic gastritis (i.e., OLGA stages I–II); sAG: patients with severe atrophic gastritis (i.e., OLGA stages III–IV); HGD/GC: patients with severe atrophic gastric and HGD or GC. Data are shown as the median, maximum, and minimum values.

The composition of the gastric bacterial community was evaluated at different taxonomic levels in both antrum and corpus biopsies. The gastric microbiota composition in *Hp*-negative patients was dominated by the Firmicutes phylum, taking up more than 50% of the relative abundance in each stage of the disease in both the antrum and the corpus. The other principal phyla observed are schematically reported in Figure 4A,B. The detailed percentages were listed in the Supplemental Materials (Table S1).

Within the most abundant phyla, thirty-three and thirty-five different classes were distinguished in both the antrum and the corpus. The most abundant ones observed belong to the Firmicutes (Bacilli, Clostridia, Negativicutes, and Erysipelotrichia classes), Proteobacteria (mostly Alphaproteobacteria and Gammaproteobacteria classes), Actinobacteria (mostly Actinobacteria and Coriobacteria classes), Fusobacteria (Fusobacteriia class), Bacteroidetes (Bacteroidia class), and Patescibacteria (Saccharimonadia class) phyla. The other microbial classes are reported in detail in the Supplemental Materials (Table S2). Differences in the amount of Clostridia were observed among groups in both the antrum and the corpus, as shown by the KW test (*p =* 0.044, *p* = 0.009, respectively). The differences were significant among CTRL and mAG compared to later stages of the disease, as shown in Figure 4C. In the corpus, the relative abundance of Negativicutes decreased throughout disease progression (Cuzick’s trend test, *p* = 0.040; *z*= −2.04, reported in Supplemental Materials). Streptococcaceae was the most abundant at the family level at each stage (Supplemental Materials Table S3). In the antrum, the relative abundance of a family belonging to the same class, Bacillaceae, was significantly higher in the HGD/GC group (post hoc result *p* < 0.001) compared to mAG patients, as shown by the post hoc results (Figure 4C).

The most abundant bacterial genus was the *Bacillus* genus, which belongs to the Bacillaceae family. The relative abundance of this genus was significantly higher in HGD/GC patients compared to both mAG and sAG, as reported in Figure 4C. Moreover, *Ralstonia* was significantly higher in mAG than in HGD/GC (Figure 4C). Differences between mAG and sAG were found among the Enterobacteriaceae family, showing significantly higher values in sAG patients exclusively in the antrum (Supplemental Materials Table S4). In the corpus, the relative abundances of the families Actinomycetaceae, Bifidobacteriaceae, and Peptostreptococcaceae were significantly higher in CTRL compared to HGD/GC and sAG patients (Figure 4C). A detailed list of bacteria genera both in the antrum and in the corpus is reported in the Supplemental Materials (Table S5).

### 3.3. Comparison of Gastric Microbial Composition in Non-Dysplastic AG and HGD/GC Patients

**Figure 5:**
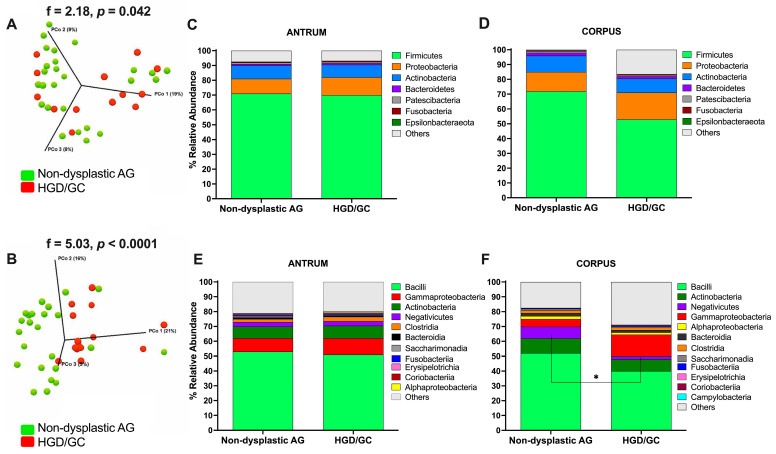
(**A**,**B**) Principal coordinate analysis (PCoA) 3D plots of the unweighted UniFrac measurements in the antrum (**A**) and the corpus (**B**). Each point represents a sample and is colored according to clinical outcome. Green dots represent non-dysplastic AG patients, and red dots represent HGD/GC patients. Statistical analyses were performed with PERMANOVA. (**C**–**F**) Bar plots represent the relative abundance of the main bacterial colonizing gastric mucosa in non-dysplastic AG and dysplastic GC in both the antrum and the corpus at the phylum (**C**,**D**) and class (**E**,**F**) levels. Data are presented as median values. Only data that show a median relative abundance higher than 0.1% are plotted. Statistical analysis comparing the relative abundance of the Negativicutes class between the two groups was performed using the Mann–Whitney U test and annotated as * *p* < 0.05. Non-dysplastic AG: patients with atrophic gastritis (mAG and sAG); HGD/GC: patients with HGD or GC.

Aiming to identify bacteria that shifted during carcinogenesis and that are potentially involved in cancer development, non-dysplastic AG patients (mild and severe AG taken together, n = 27) and HGD/GC patients (n = 14) were compared for a total of 41 patients. As shown by the PERMANOVA results of comparing non-dysplastic AG and HGD/GC patients, the differences in microbial composition between the two groups were significant for all the beta diversity indices considered (Supplemental Materials Table S6). In the PCoA graphs in Figure 5A,B, two distinct populations were distinguished, indicating the marked differentiation of the two clusters. In both the antrum and the corpus, Firmicutes was the most abundant phylum in each group, followed by Proteobacteria, Actinobacteria, Bacteroidetes, Patescibacteria, and Fusobacteria (Figure 5C,D). No significant differences were found at the phylum level. At the class level, the abundance order differed between the antrum and the corpus (Figure 5E,F). No significant differences were found at the class level in the antrum. In the corpus, the most abundant class was Bacilli (52.0% and 40.0% median values in non-dysplastic AG and HGD/GC, respectively), Actinobacteria (10.0% and 8.0%, respectively), Negativicutes (8.0% and 2.0%, respectively), Gammaproteobacteria (5.0% and 14.5%, respectively), Alphaproteobacteria (2.0% and 1.5%, respectively), Bacteroidia (2.0% and 1.5%, respectively), and Clostridia (2.0% for both groups). A significant reduction in the percentage of Negativicutes was found in HGD/GC patients (MW, *p* = 0.045), as reported in Figure 5F.

**Figure 6:**
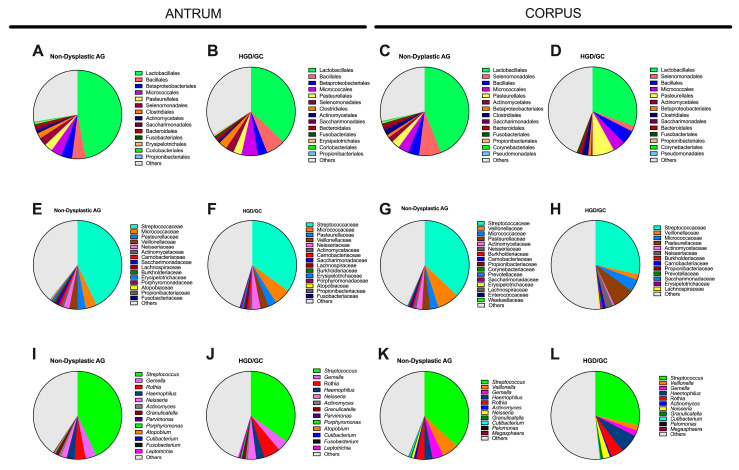
Pie charts representing the difference in the relative abundance of the main bacterial orders (**A**–**D**), families (**E**–**H**), and genera (**I**–**L**) colonizing gastric tissue in both the antrum and the corpus, comparing non-dysplastic AG patients and AG patients with high-grade dysplasia or GC. Data are presented as median values. Only data showing a median relative abundance value higher than 0.001% are reported.

**Figure 7:**
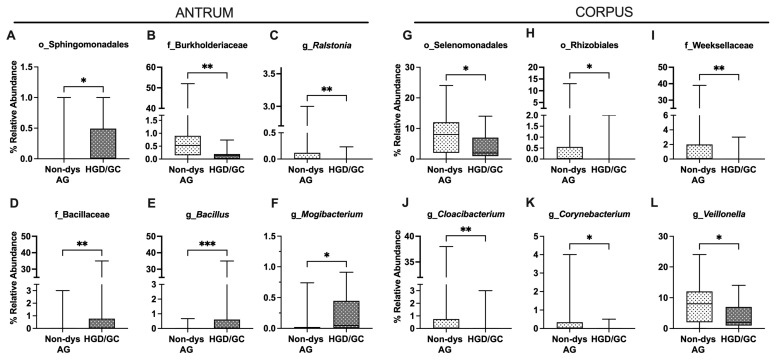
Relative abundance comparison of gastric bacteria significantly differed between non-dysplastic AG (non-dys AG) and patients who progressed through dysplasia and cancer (HGD/GC) both in the antrum (**A**–**F**) and in the corpus (**G**–**L**). (p, phylum; c, class; o, order; f, family; g, genus). Data are presented as the median, minimum, and maximum values. Statistical differences in the relative abundance were analyzed using the Mann–Whitney U test and annotated as * *p* < 0.05, ** *p* < 0.01, *** *p* < 0.001.

Bacterial relative abundance at lower taxonomic levels is reported in Figure 6. In the antrum, the relative abundance of the Sphingomonadales order (Alphaproteobacteria class) was higher in HGD/GC patients than in non-dysplastic AG patients (Figure 7A). The decrease in the Burkholderiaceae family and its *Ralsonia* genus in HGD/GC patients was significant (Figure 7B,C). On the contrary, a significant increase in Bacillaceae (and the *Bacillus* genus) percentage in HGD/GC patients compared to in non-dysplastic AG patients (Figure 7D,E) was observed. Moreover, *Mogibacterium* abundance was higher in HGD/GC patients (Figure 7F). In the HGD/GC group, a significant reduction in the percentage of Selenomonadales and Rhizobiales orders in the corpus was observed (Figure 7G,H). At the family level, the relative percentage of the Weeksellaceae (belonging to the Flavobacteriales order) and its genus *Cloacibacterium* were significantly lower in HGD/GC patients, as shown in Figure 7I,J. Moreover, the abundance of the *Corynebacterium* and *Veillonella* genera (belonging to the Selenomonadales order) decreased significantly in the HGD/GC group (Figure 7K,L).

### 3.4. Development of a Risk Score Based on Multi-Microbial Parameters

The possible association between microbial composition and cancer progression was investigated using a logistic regression analysis (univariate), including all the significant bacterial features observed in the previous analyses as independent variables.

Considering the low relative abundance of these bacteria potentially associated with cancer development, the presence of these bacteria was converted into a dummy variable (1 = presence, 0 = absence). As fully described in Table 3, in the antrum, the Bacillaceae family was observed in less than 30.0% of AG patients and in more than 70.0% of HGD/GC patients (8 out of 27 AG patients versus 10 out of 14 HGD/GC patients). Within this family, the percentage of *Bacillus* increased, and its prevalence in HGD/GC patients was more than 70.0%. The logistic regression analysis showed a significant increase in the risk of cancer development for both Bacillaceae and *Bacillus* presence in the antrum (odds ratio [OR] 5.94, *p* = 0.019; OR 14.38, *p* < 0.001, respectively). Remaining within the antrum, the prevalence of *Ralstonia* was higher in the non-dysplastic AG (52.0% in non-dysplastic AG versus 7.0% in HGD/GC patients). The absence of *Ralstonia* was related to a higher risk of developing cancer (OR = 14.00, *p* = 0.006). In the corpus, the Rhizobiales order, Weeksellaceae family, and *Cloacibacterium* genus significantly decreased in relative abundance (Table 3). The bacteria were present in just over 20.0% of HGD/GC patients and, on the contrary, in more than 70.0% of non-dysplastic AG patients. The *Veillonella* genus was also reduced in HGD/GC patients (Table 3). The univariate analysis showed that the loss of these bacteria in HGD/GC patients was correlated to an increased risk of cancer development, as shown in Table 3 (OR = 8.94, *p* = 0.048).

All seven significant differences included in the first step of the univariate logistic regression analysis were further considered for a multivariate analysis, reported in Table 3. Among the seven differences, just four remained significant after the second step of multivariate regression analysis, which significantly distinguished non-dysplastic AG from HGD/GC patients. The four features were considered as possible risk conditions for the development of GC. Based on these results, the risk conditions were used to design a risk score.

**Figure 8:**
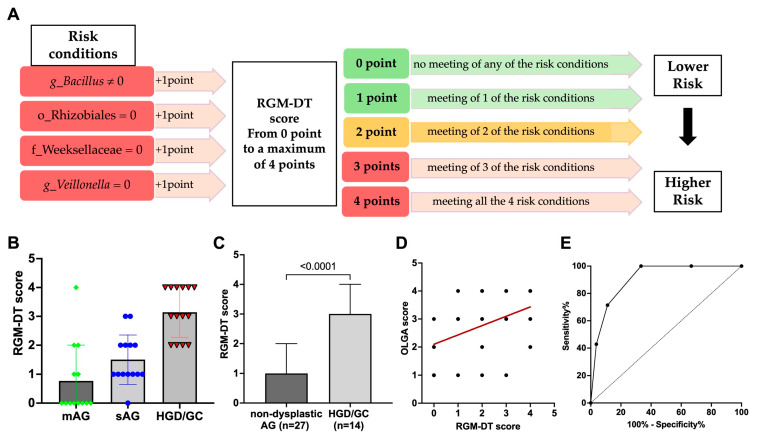
(**A**) Schematic representation of points assigned to each patient to construct the proposed RGM-DT scoring system. (p, phylum; c, class; o, order; f, family; g, genus). (**B**) RGM-DT score for each patient according to the disease group. (**C**) RGM-DT score in the non-dysplastic AG group (mild and severe AG taken together) compared to the dysplastic AG group. Data are reported as mean and standard deviation. Statistical analysis was performed using the Mann–Whitney U test. (**D**) Correlation between RGM-DT and OLGA score. (**E**) The ROC curve analysis shows the performance of the RGM-DT score in discriminating between non-dysplastic AG patients and HGD/GC AG patients.

To calculate a risk score, one point was assigned for possessing each risk condition, as schematically reported in Figure 8A. Accordingly, each patient could have a final score from a minimum value of zero (no meeting of any risk condition, corresponding to a lower risk) to a maximum value of four (all conditions met, corresponding to a higher risk of progressing through dysplasia). The final score was calculated by adding up every single result and is called the “Resident Gastric Microbiota Dysbiosis Test” (RGM-DT). Each patient’s value is reported in Figure 8B. The mean value of the RGM- DT score was 3.14 (±0.90) in AG patients with HGD/GC and 1.15 (±1.10) in AG patients without dysplasia (MW, *p* < 0.0001) (Figure 8C). In the mAG group, 9 out of 13 patients (about 70.0%) had a score equal to zero; 2 patients (15.0%) showed a score equal to one. In the same group, no patients had an RGM-DT score equal to three, and one patient showed a score equal to four. In the sAG group, 5 out 14 patients (36.0%) had an RGM-DT score equal to zero or one, 1 patient showed a score equal to two, and 3 patients (21.0%) had a score equal to three. In the HGD/GC group, no patient had an RGM-DT score equal to zero, 14.0% of patients (two out of 14) showed a score equal to one, and the same percentage was recorded for patients with a score equal to two. Four patients out of 14 (29.0%) had an RGM-DT equal to three, and 6 patients (43.0%) had a score equal to four. Moreover, the RGM-DT score was positively correlated to atrophy grade (Spearman’s rank correlation coefficient, r_s_ = +0.44, *p* = 0.004; Figure 8D).

The performance of the RGM-DT in identifying the HGD/GC patients from non-dysplastic AG patients was satisfactory, with accuracy based on the area under the curve (AUC = 0.907 ± 0.045) in our cohort. The ROC analysis also indicated that an RGM-DT ≥ 3 would maximize the sensitivity and specificity, yielding the best combination (sensitivity 71.4%, [95% CI 45.3–88.3]) and specificity (88.9% [95% CI 71.9–96.1]), and a negative predicted value of 86.0% and an accuracy of 83.0%. The results are presented in Figure 8E. Accordingly, with a result equal to or higher than 3, the test was considered positive and the patient was considered “high risk.” On the contrary, with a score lower than 3, the test was considered negative. Applying this cut-off, 82.9% of patients were correctly classified. During the medium follow-up time of 84 months, none of the AG patients with a negative RGM-DT developed dysplasia or cancer.

## 4. Discussion

GC is nowadays a significant cause of death worldwide. In Italy, the mortality is still high and the development of new methods to increase early detection remains fundamental. Despite *Hp* infection being the principal risk factor for GC, several studies have shown a decrease in *Hp* abundance in the gastric mucosa of GC patients and demonstrated a microbial imbalance in the gastric microbiota during carcinogenesis regardless of *Hp* infection [13,19,25]. Nevertheless, the potential role of other bacteria during GC development is largely unknown.

Our work, analyzing gastric microbiota composition in European *Hp*-negative patients, was focused on identifying bacteria composition changes along with GC pathogenesis in both the antrum and the corpus.

The goal was to develop a multiparametric score that could be used in clinical practice to improve patient management by predicting the risk of progression towards GC in patients affected by AG. With these aims, the relative abundance of bacteria colonizing gastric mucosa in patients without any gastric disease (controls), in patients affected by AG with OLGA I–II staging and AG with OLGA III–IV staging, and in patients affected by severe AG and HGD or gastric cancer was compared.

Contrary to several data obtained from *Hp*-positive patients, the gastric microbiota composition in *Hp*-negative patients was dominated by the Firmicutes phylum, taking up more than 50% of the relative abundance in each stage of the disease both in the antrum and in the corpus mucosa. These results are similar to previous findings [23,26,27,28,29]. Alpha diversity measures showed that healthy patients were characterized by a higher bacterial diversity and richness compared to AG and cancer patients, whereas other groups were similar [13,30,31]. Therefore, this resulted in a loss of microbial diversity during carcinogenesis. Beta diversity results showed significant differences between CTRL, mAG, sAG and HGD/GC patients, especially in the corpus, suggesting that other modifications of mucosal microbiota occurred during GC progression. Differences in bacterial composition between mAG and sAG were also observed. In particular, the abundance of Enterobacteriaceae was higher in sAG compared to mAG. Enterobacteriaceae are related to inflammation [32], and their increase was reported in both the premalignant gastric stage and cancer [33]. The percentage of Burkholderiaceae was also different among groups; in particular, it was higher in sAG compared to CTRL and mAG patients. Bacteria belonging to the Burkholderiaceae family were previously reported as responsible for mitogen-activated protein kinases/extracellular signal-regulated kinase (MAPK/ERK) pathway activation [32]. Thus, the higher level of both Enterobacteriaceae and Burkholderiaceae could influence cancer development by increasing cell proliferation, migration, and apoptosis inhibition during the sAG stage. The relative abundance of the Clostridia class was different during disease progression in both the antrum and corpus. This class of bacteria is typical of gut flora, and its presence is fundamental for intestinal homeostasis [34,35]. Conversely, from another study [19], our results showed that, in the antrum, the amount of Clostridia was higher in CTRL and mAG compared to sAG. In the corpus, this difference in Clostridia class abundance was recognizable in the decreased amount in the Peptostreptococcaceae family. Although a relationship between this family and colorectal cancer has been previously observed [36], no deeper investigations were performed in GC, suggesting the need for further studies. Moreover, in the corpus microbiome, Actinomycetaceae and Bifidobacteriaceae families, both belonging to the Actinomycetales order, were decreased. Similar data were reported by Gunathilake, who investigated healthy screened subjects and early GC patients [37].

Further analyses compared AG patients without dysplasia (mild and severe AG considered together) and AG patients with HGD to highlight bacteria possibly involved in GC onset. Interestingly, significant differences in beta diversity were observed by comparing these two groups, as shown in the PERMANOVA results of both the antrum and the corpus. The MW test was performed to compare the differences in the relative abundance of bacteria inhabiting the antrum between non-dysplastic AG and patients with HGD/GC. Bacteria belonging to the Sphingomonadales order and the Bacillaceae family significantly increased during cancer development. A previous study did not identify Bacillaceae as a bacteria contributing to gastric carcinogenesis, suggesting instead that it could be helpful as an antagonistic and potentially a probiotic [38]. On the contrary, similarly to another study, our data showed an increased level of Bacilli in GC patients [37]. The bacteria belonging to this family exhibit nitrate and nitrite reductase activity [39], which is generally upregulated in the resident microbiota of patients affected by GC [19,40]. The over-representation of these bacteria in HGD/GC could contribute, together with the other factors, to the pro-inflammatory condition that leads to cancer development. A higher abundance of the *Mogibacterium* genus was also observed. This bacterium colonizes oral mucosa, and its overabundance in the antrum could be imputable to a higher pH condition of the stomach [41,42]. The atrophy and the loss of the glandular tissue in the stomach result in decreased acid secretion and, therefore, it could increase intestinal commensals and oral species [11,43,44]. Oral bacteria were also associated with other diseases, including colorectal and pancreatic cancer [20]. On the contrary, bacteria belonging to the Burkholderiaceae (*Ralstonia* genus) and Corynebacteriaceae families significantly decreased throughout disease progression. Similarly to other studies [45,46], *Ralstonia* abundance increased at the initial stage of the disease and then decreased, as shown in our data. This could be explained because *Ralstonia* has been recognized as a player in starting inflammation, but it was probably replaced by other bacteria later in the disease stage. In the corpus sample, the reduction in bacteria belonging to the Selenomonadales and Rhizobiales orders, the Weeksellaceae family, and the *Corynebacterium* genus were significant during cancer progression.

It has already been demonstrated that cancer surveillance can be improved by introducing the detection of specific bacteria within screening or follow-up protocols, increasing the diagnostic procedure’s performance [11,47,48]. To find specific parameters of gastric microbiota that can be used for this aim, here we considered the low-abundance bacteria that showed a different prevalence in the gastric mucosa to distinguish HGD/cancer patients from AG patients without HGD/cancer. Data obtained with this analysis were used to develop the RGM-DT, and its performance was verified with an ROC curve. The RGM-DT could be a potential diagnostic marker for the early detection of GC, as demonstrated by an AUC of 0.907 in our discovery cohort. Interestingly, during the medium follow-up time of 84 months, none of the AG patients with a negative RGM-DT developed dysplasia or cancer.

Our data differed slightly from other papers, even though the same type of samples were analyzed. An explanation could be found in the difference in lifestyle and eating habits of our cohort of Europeans compared to patients of other studies on this topic, often including *Hp*-positive patients [49]. Moreover, differences in our data could also be due to the separate analysis of the antrum and corpus. Considering that similar gastric microbiota composition should be found in the antrum and corpus, some papers analyzed them without distinction [18,26,50]. However, another study recommends the separation of the antrum and corpus to obtain a comprehensive view of gastric microbiota [51]. Overall, our analysis underlines that the stomach microbiota is not uniform, suggesting that it could be helpful to discriminate between the antrum and corpus sites when microbiota composition is investigated.

This study has some limitations. The risk model described is based on data from a single center and includes a quite low sample size. The presented test’s real clinical impact and performance need to be validated in an independent, larger validation cohort of patients with a longer follow-up. Moreover, the microbial composition evaluation based on the sequencing of 16rRNA shows low power at the species level.

## 5. Conclusions

Most descriptive papers reporting the composition of gastric microbiota in patients with different gastric diseases are mainly not focused on the clinical practical usefulness of this kind of information. The presented data suggest that analyzing gastric microbiota could help stratify cancer risk in patients with atrophic gastritis. We have developed a simple predictive risk score that shows good performance in clustering patients with HGD or gastric cancer, offering the possibility to help clinicians in daily practice. The model described here was built in a European *Hp*-negative population representing the gastric microbiota composition of AG patients commonly seen in Europe. After validation in a larger cohort, the Resident Gastric Microbiota Dysbiosis Test could be integrated into our healthcare system to avoid expensive close follow-up endoscopies in low-risk patients. Finally, we speculated on the possibility of using prebiotics/probiotics or food intervention, as has already been observed by other works [52,53]. Further investigation will be interesting on using prebiotics/probiotics or food intervention to “revert” a dysbiotic situation within a more cost-effective and personalized follow-up program.

## Figures and Tables

**Table 1:** Characteristics of patients in our cohort according to each group.

	CTRL	mAG	sAG	HGD/GC	*p*-Value
n of patients	11	13	14	14	n.a.
AGE mean (±SD)	66.4 (±10.1)	69.9 (±10.2)	66.3 (±12.9)	76.1 (±8.6)	0.375 *
MALE n (%)	9 (81.8)	7 (53.9)	5 (35.7)	10 (71.4)	n.a.
FEMALE n (%)	2 (18.2)	6 (46.1)	9 (64.3)	4 (28.6)	
OTU mean (±SD) antrum	437.2 (±90.7)	404.3 (±145.3)	391.4 (±95.9)	309.4 (±132.0)	0.056 *
OTU mean (±SD) corpus	399.8 (±120.8)	420.2 (±100.6)	425.4 (±134.7)	280.6 (±78.5)	0.003 *

Data are shown as the mean and standard deviation (±SD). CTRL are healthy subjects; mAG: patients with mild atrophic gastritis (i.e., OLGA stages I–II); sAG: patients with severe atrophic gastritis (i.e., OLGA stages III–IV); HGD/GC: patients with severe AG and HGD or GC. * ANOVA. n.a.: not applicable.

**Table 2:** PERMANOVA analysis: comparison of beta diversity among groups studied based on the unweighted UniFrac matrix.

PERMANOVA Analysis in the Antrum f-Statistic 1.96, *p*-Value = 0.010			PERMANOVA Analysis in the Corpus f-Statistic 3.97, *p*-Value < 0.001		
**GROUP**	**GROUP**	***p-*** Value *	**GROUP**	**GROUP**	***p-*** Value *
CTRL	mAG	n.s.	CTRL	mAG	0.022
CTRL	HGD/GC	0.010	CTRL	HGD/GC	<0.001
mAG	sAG	n.s.	mAG	sAG	0.002
mAG	HGD/GC	n.s.	mAG	HGD/GC	0.047
sAG	HGD/GC	n.s.	sAG	HGD/GC	0.026
CTRL	sAG	0.009	CTRL	sAG	0.007

* The *p*-value obtained with PERMANOVA is adjusted by Bonferroni correction. n.s.: *p*-value is not statistically significant (*p* > 0.05). CTRL: healthy subjects; mAG: patients with mild atrophic gastritis (i.e., OLGA stages I–II); sAG: patients with severe atrophic gastritis (i.e., OLGA stages III–IV); HGD/GC: patients with severe atrophic gastric and HGD or GC.

**Table 3:** The univariate and multivariate binary logistic regression analyses according to the risk conditions for GC progression.

							Univariate			Multivariate	
	Bacteria	Group	Presence	%	Absence	%	OR	95% CI	*p*-Value	*p*-Value	95% CI
**Antrum**	f_Bacillaceae	Non-dysplastic AG (27)	8	29.6	19	70.4	5.94	1.44 to 20.29	0.019	n.s.	
		HGD/GC (14)	10	71.4	4	28.6					
	*g_Bacillus*	Non-dysplastic AG (27)	4	14.8	23	85.2	14.38	2.80 to 64.74	*p* < 0.001	0.001	0.20 to 0.66
		HGD/GC (14)	10	71.4	4	28.6					
	*g_Ralstonia*	Non-dysplastic AG (27)	14	51.9	13	48.1	14.00	1.73 to 158.30	0.006	n.s.	
		HGD/GC (14)	1	7.1	13	92.9					
**Corpus**	o_Rhizobiales	Non-dysplastic AG (27)	19	70.4	8	29.6	5.94	1.44 to 20.29	0.019	0.007	0.09 to 0.55
		HGD/GC (14)	4	28.6	10	71.4					
	f_Weeksellaceae	Non-dysplastic AG (27)	21	77.8	6	22.2	12.83	2.62 to 49.94	0.001	0.032	0.25 to 0.52
		HGD/GC (14)	3	21.4	11	78.6					
	*g_Cloacibacterium*	Non-dysplastic AG (27)	20	74.1	7	25.9	10.48	2.23 to 40.32	0.002	n.s.	
		HGD/GC (14)	3	21.4	11	78.6					
	*g_Veillonella*	Non-dysplastic AG (27)	11	40.7	16	59.3	8.94	1.02 to 78.59	0.048	0.040	0.03 to 0.44
		HGD/GC (14)	1	7.1	13	92.9					

Data are reported as the number and the percentage of patients showing the presence or the absence of each bacterium considered. n.s.: *p*-value is not statistically significant (*p* > 0.05). (p, phylum; c, class; o, order; f, family; g, genus).

## Data Availability

All data relevant to the study are included in the article or uploaded as Supplementary Materials.

## Supplementary Materials

The following supporting information can be downloaded at: https://www.mdpi.com/article/10.3390/nu17010142/s1. Relative abundance values and statistical analyses of the main bacteria phyla (Table S1), classes (Table S2), orders (Table S3), families (Table S4), and genera (Table S5) colonizing the gastric antrum (above) and the corpus (below) in descending order of abundance in each group. Table S6. PERMANOVA analysis results according to different beta diversity indices. Comparison of beta diversity between non-dysplastic AG and dysplastic/cancer AG patients in both antrum and corpus microbial composition. Figure S1. Comparison of the number of OTUs between the antrum and the corpus at each stage of the disease.
