# Supplementary material for: Predictive Value of a Gastric Microbiota Dysbiosis Test for Stratifying Cancer Risk in Atrophic Gastritis Patients
Source: Nutrients. 2024 Dec 31;17(1):142. doi: 10.3390/nu17010142 (PMC11722812; doi:10.3390/nu17010142)
Supplement: Supplementary file 1 [file nutrients-17-00142-s001.zip › nutrients-3379337-supplementary.pdf]

SUPPLEMENTAL MATERIALS

Table S1. Relative abundance values and statistical analyses of the main bacteria phyla colonizing the gastric antrum (above) and corpus (below) in descending order of abundance in each group. Data are expressed as the median, first quartile (Q1) and third quartile (Q3). In the tables are reported the phyla with a percentage higher than 0.05%. KW represents the *p values* resulting from the Kruskal-Wallis test. Cuzick test for trend is expressed with the *p value* and, if significant, with the z value of trend. *p values* are annotated as *p<0.05. n.s. represent a *p value* non-significant (p> 0.05). CTRL are patients with any gastric disease; mAG: patients with moderate atrophic gastritis (i.e. OLGA stage I-II); sAG: patients with severe atrophic gastritis (i.e. OLGA stage III-IV); HGD/GC: patients with severe atrophic gastritis and high-grade dysplasia or GC.

ANTRUM	CTRL			mAG			sAG			HGD/GC			KW	CUZICK	
	Median	Q1	Q3	Median	Q1	Q3	Median	Q1	Q3	Median	Q1	Q3	*p*	*p*	z
Firmicutes	57.000	53.000	64.500	73.000	55.000	77.000	62.000	44.250	78.500	70.000	52.500	83.250	n.s.	n.s.	/
Proteobacteria	21.000	12.000	31.500	10.000	4.000	23.000	12.500	6.000	33.500	12.000	5.750	23.250	n.s.	n.s.	/
Actinobacteria	10.000	7.500	12.500	10.000	4.000	12.000	8.500	6.250	12.750	8.500	6.250	11.000	n.s.	n.s.	/
Fusobacteria	3.000	0.661	4.500	0.560	0.169	1.000	0.794	0.025	2.000	0.706	0.083	3.000	n.s.	n.s.	/
Bacteroidetes	2.000	0.844	5.500	2.000	0.611	2.000	0.841	0.202	4.500	1.000	0.588	2.000	n.s.	n.s.	/
Patescibacteria	2.000	1.000	3.000	2.000	0.217	5.000	0.770	0.009	3.000	0.924	0.078	1.000	n.s.	*	-1.96
Epsilonbacteraeota	0.370	0.001	0.617	0.000	0.000	0.001	0.000	0.000	0.124	0.000	0.000	0.047	n.s.	*	-2.21

CORPUS	CTRL			mAG			sAG			HGD/GC			KW	CUZICK	
	Median	Q1	Q3	Median	Q1	Q3	Median	Q1	Q3	Median	Q1	Q3	*p*	*p*	z
Firmicutes	54.000	44.000	69.000	72.000	55.000	78.000	67.000	49.500	77.750	53.000	36.250	64.750	n.s.	n.s.	/
Proteobacteria	15.000	6.000	22.000	13.000	5.000	22.000	7.500	4.000	23.750	18.000	10.500	28.000	n.s.	n.s.	/
Actinobacteria	14.000	8.000	20.500	11.000	3.000	11.000	11.000	6.250	15.500	9.500	3.250	16.750	n.s.	n.s.	/
Bacteroidetes	2.000	0.513	10.500	4.000	2.000	7.000	0.994	0.058	5.750	1.500	0.073	3.500	n.s.	n.s.	/
Patescibacteria	2.000	1.000	4.500	1.000	0.008	2.000	1.500	0.448	2.750	0.917	0.235	2.750	n.s.	n.s.	/
Fusobacteria	0.075	0.004	3.000	0.894	0.005	1.000	0.396	0.006	1.750	0.671	0.124	2.750	n.s.	n.s.	/
Epsilonbacteraeota	0.056	0.000	0.636	0.000	0.000	0.000	0.000	0.000	0.003	0.040	0.000	0.335	n.s.	n.s.	/

Table S2. Relative abundance values and statistical analyses of the main bacteria classes colonizing the gastric antrum (above) and corpus (below) in descending order of abundance in each group. Data are expressed as the median, first quartile (Q1) and third quartile (Q3). In the tables are reported the classes with a percentage higher than 0.05%. KW represents the p values resulting from the Kruskal-Wallis test. Cuzick test for trend is expressed with the *p value* and, if significant, with the z value of trend. *p values* are annotated as * p<0.05,** p<0.01. n.s. represent a *p value* non-significant (p> 0.05). CTRL are patients with any gastric disease; mAG: patients with moderate atrophic gastritis (i.e. OLGA stage I-II); sAG: patients with severe atrophic gastritis (i.e. OLGA stage III-IV); HGD/GC: patients with severe atrophic gastritis and high-grade dysplasia or GC.

ANTRUM	CTRL			mAG			sAG			HGD/GC			KW	CUZICK	
	Median	Q1	Q3	Median	Q1	Q3	Median	Q1	Q3	Median	Q1	Q3	*p*	*p*	z
Bacilli	37.000	30.000	52.000	57.000	47.000	71.000	51.500	37.000	65.000	51.000	38.250	75.000	n.s.	n.s.	/
Gammaproteobacteria	19.000	5.000	31.000	8.000	4.000	22.000	12.000	4.250	33.000	11.000	4.500	22.500	n.s.	n.s.	/
Actinobacteria	8.000	5.500	10.500	8.000	4.000	11.000	7.500	5.250	12.750	8.500	5.250	10.750	n.s.	n.s.	/
Negativicutes	5.000	1.500	13.000	2.000	0.832	7.000	4.000	3.000	8.000	3.000	1.250	5.500	n.s.	n.s.	/
Clostridia	4.000	3.000	10.500	5.000	2.000	8.000	2.000	0.611	2.750	3.000	2.250	5.000	*	n.s.	/
Fusobacteriia	3.000	0.661	4.500	0.560	0.169	1.000	0.794	0.025	2.000	0.706	0.083	3.000	n.s.	n.s.	/
Saccharimonadia	2.000	1.000	3.000	2.000	0.123	4.000	0.770	0.008	3.000	0.556	0.048	1.000	n.s.	n.s.	/
Erysipelotrichia	1.000	0.509	2.000	0.490	0.007	2.000	0.575	0.007	1.000	0.545	0.008	0.966	n.s.	n.s.	/
Coriobacteriia	0.807	0.300	2.500	0.004	0.002	2.000	0.348	0.020	0.806	0.002	0.000	0.614	n.s.	*	-2.58
Alphaproteobacteria	0.739	0.227	1.438	0.313	0.152	1.000	0.510	0.015	0.986	0.876	0.130	2.750	n.s.	n.s.	/
Campylobacteria	0.370	0.001	0.617	0.000	0.000	0.001	0.000	0.000	0.124	0.000	0.000	0.047	n.s.	*	-2.21

CORPUS	CTRL			mAG			sAG			HGD/GC			KW	CUZICK	
	Median	Q1	Q3	Median	Q1	Q3	Median	Q1	Q3	Median	Q1	Q3	*p*	*p*	z
Bacilli	36.000	20.500	51.500	52.000	46.000	59.000	50.000	33.750	71.250	40.000	28.500	57.000	n.s.	n.s.	/
Actinobacteria	13.000	7.500	18.000	9.000	3.000	11.000	10.000	6.000	15.500	8.000	3.000	16.750	n.s.	n.s.	/
Gammaproteobacteria	7.000	5.000	18.000	7.000	4.000	17.000	5.000	3.000	20.500	14.500	5.750	24.750	n.s.	n.s.	/
Clostridia	6.000	4.500	6.500	2.000	1.000	4.000	1.500	0.546	2.750	2.000	1.226	3.000	**	*	-2.51
Negativicutes	4.000	2.000	19.000	8.000	3.000	10.000	6.000	2.000	11.750	2.000	1.000	6.500	n.s.	*	-2.04
Bacteroidia	2.000	0.513	10.500	4.000	2.000	7.000	0.994	0.058	5.750	1.500	0.073	3.500	n.s.	n.s.	/
Saccharimonadia	2.000	1.000	4.000	0.947	0.005	2.000	1.500	0.448	2.000	0.566	0.228	2.500	n.s.	n.s.	/
Alphaproteobacteria	1.000	0.449	5.500	2.000	1.000	9.000	0.432	0.135	2.000	1.500	0.244	4.000	n.s.	n.s.	/
Coriobacteriia	1.000	0.388	1.000	0.006	0.000	0.228	0.005	0.000	0.732	0.001	0.000	0.557	*	*	-2.22
Erysipelotrichia	1.000	0.645	3.000	0.044	0.001	1.000	0.329	0.001	2.000	0.410	0.001	1.000	n.s.	n.s.	/
Fusobacteriia	0.075	0.004	3.000	0.894	0.005	1.000	0.396	0.006	1.750	0.671	0.124	2.750	n.s.	n.s.	/

Table S3. Relative abundance values and statistical analyses of the main bacteria orders colonizing the gastric antrum (above) and corpus (below) in descending order of abundance in each group. Data are expressed as the median, first quartile (Q1) and third quartile (Q3). In the tables are reported the orders with a percentage higher than 0.001%. KW represents the *p value*s resulting from the Kruskal-Wallis test. Cuzick test for trend is expressed with the *p value* and, if significant, with the z value of trend. *p values* are annotated as *p<0.05,** p<0.01. n.s. represent a *p value* non-significant (p> 0.05). CTRL are patients with any gastric disease; mAG: patients with moderate atrophic gastritis (i.e. OLGA stage I-II); sAG: patients with severe atrophic gastritis (i.e. OLGA stage III-IV); HGD/GC: patients with severe atrophic gastritis and high-grade dysplasia or GC.

ANTRUM	CTRL			mAG			sAG			HGD/GC			KW	CUZICK	
	Median	Q1	Q3	Median	Q1	Q3	Median	Q1	Q3	Median	Q1	Q3	*p*	*p*	z
Lactobacillales	33.000	23.000	42.500	47.000	38.000	62.000	48.500	32.500	53.000	37.000	28.500	61.250	n.s.	n.s.	/
Pasteurellales	10.000	2.000	12.500	2.000	0.257	8.000	5.000	0.532	13.000	3.000	1.186	11.500	n.s.	n.s.	/
Betaproteobacteriales	9.000	0.530	18.000	4.000	1.000	9.000	3.500	0.701	15.000	3.500	0.894	7.500	n.s.	n.s.	/
Bacillales	8.000	2.500	11.000	6.000	2.000	9.000	3.500	2.000	6.000	7.000	3.500	9.750	n.s.	n.s.	/
Micrococcales	5.000	1.500	7.500	3.000	1.000	7.000	5.000	3.000	10.500	6.000	4.000	9.750	n.s.	n.s.	/
Selenomonadales	5.000	1.500	13.000	2.000	0.832	7.000	4.000	3.000	8.000	3.000	1.250	5.500	n.s.	n.s.	/
Clostridiales	4.000	3.000	10.500	5.000	2.000	8.000	2.000	0.611	2.750	3.000	2.250	5.000	*	n.s.	/
Fusobacteriales	3.000	0.661	4.500	0.560	0.169	1.000	0.794	0.025	2.000	0.706	0.083	3.000	n.s.	n.s.	/
Actinomycetales	2.000	2.000	3.000	1.000	0.635	3.000	1.500	0.706	3.000	0.911	0.516	1.000	n.s.	*	-2.25
Saccharimonadales	2.000	1.000	3.000	2.000	0.123	4.000	0.770	0.008	3.000	0.556	0.048	1.000	n.s.	n.s.	/
Bacteroidales	1.000	0.509	4.500	2.000	0.207	2.000	0.590	0.010	3.250	0.782	0.490	0.995	n.s.	n.s.	/
Erysipelotrichales	1.000	0.509	2.000	0.490	0.007	2.000	0.575	0.007	1.000	0.545	0.008	0.966	n.s.	n.s.	/
Coriobacteriales	0.807	0.300	2.500	0.004	0.002	2.000	0.348	0.020	0.806	0.002	0.000	0.614	n.s.	*	-2.58
Campylobacterales	0.370	0.001	0.617	0.000	0.000	0.001	0.000	0.000	0.124	0.000	0.000	0.047	n.s.	*	-2.21
Corynebacteriales	0.160	0.004	0.641	0.343	0.001	1.000	0.023	0.000	0.222	0.045	0.002	0.233	n.s.	n.s.	/
Propionibacteriales	0.149	0.070	0.280	0.060	0.001	0.255	0.296	0.053	0.381	0.143	0.055	0.521	n.s.	n.s.	/
Sphingomonadales	0.146	0.001	0.430	0.000	0.000	0.000	0.000	0.000	0.001	0.005	0.000	0.429	**	*	/
Pseudomonadales	0.091	0.001	0.677	0.271	0.002	0.397	0.001	0.000	0.180	0.083	0.000	0.224	n.s.	n.s.	/
Flavobacteriales	0.083	0.023	0.110	0.005	0.002	0.339	0.002	0.000	0.110	0.416	0.007	0.679	n.s.	n.s.	/
Bifidobacteriales	0.006	0.000	0.116	0.000	0.000	0.002	0.000	0.000	0.142	0.000	0.000	0.004	n.s.	n.s.	/
Enterobacteriales	0.001	0.000	0.019	0.000	0.000	0.000	0.023	0.000	0.363	0.000	0.000	0.001	n.s.	n.s.	/

CORPUS	CTRL			mAG			sAG			HGD/GC			KW	CUZICK	
	Median	Q1	Q3	Median	Q1	Q3	Median	Q1	Q3	Median	Q1	Q3	*p*	*p*	z
Lactobacillales	29.000	17.500	35.500	44.000	31.000	52.000	45.500	30.250	59.000	30.500	22.500	49.500	n.s.	n.s.	/
Bacillales	6.000	3.000	10.500	11.000	4.000	15.000	3.500	3.000	4.750	5.000	2.250	9.750	n.s.	n.s.	/
Clostridiales	6.000	4.500	6.500	2.000	1.000	4.000	1.500	0.546	2.750	2.000	1.226	3.000	**	*	-2.51
Pasteurellales	4.000	2.000	7.500	2.000	0.010	3.000	3.000	1.000	8.750	8.000	0.710	12.000	n.s.	n.s.	/
Selenomonadales	4.000	2.000	19.000	8.000	3.000	10.000	6.000	2.000	11.750	2.000	1.000	6.500	n.s.	*	-2.41
Actinomycetales	3.000	2.500	6.000	2.000	0.607	2.000	0.810	0.574	3.000	0.593	0.240	2.000	*	*	-2.54
Betaproteobacteriales	3.000	0.564	10.500	2.000	1.000	5.000	1.500	0.972	6.500	3.000	2.000	7.500	n.s.	n.s.	/
Micrococcales	3.000	2.000	4.000	2.000	0.210	6.000	5.000	3.000	8.500	4.500	2.000	11.750	n.s.	n.s.	/
Bacteroidales	2.000	0.101	6.500	2.000	0.006	3.000	0.634	0.005	1.750	1.000	0.073	2.000	n.s.	n.s.	/
Saccharimonadales	2.000	1.000	4.000	0.947	0.005	2.000	1.500	0.448	2.000	0.566	0.228	2.500	n.s.	*	-2.06
Coriobacteriales	1.000	0.388	1.000	0.006	0.000	0.228	0.005	0.000	0.732	0.001	0.000	0.557	*	*	-2.22
Erysipelotrichales	1.000	0.645	3.000	0.044	0.001	1.000	0.329	0.001	2.000	0.410	0.001	1.000	n.s.	n.s.	/
Corynebacteriales	0.573	0.182	2.000	0.731	0.003	1.000	0.305	0.038	0.756	0.004	0.000	0.691	n.s.	*	-2.16
Bifidobacteriales	0.490	0.005	1.500	0.001	0.000	0.002	0.009	0.000	0.128	0.000	0.000	0.000	*	**	-2.87
Propionibacteriales	0.462	0.196	2.500	0.878	0.360	3.000	0.460	0.075	0.865	0.182	0.073	1.750	n.s.	n.s.	/
Pseudomonadales	0.083	0.024	0.287	0.634	0.025	2.000	0.001	0.000	0.157	0.018	0.000	0.166	*	*	-2.11
Fusobacteriales	0.075	0.004	3.000	0.894	0.005	1.000	0.396	0.006	1.750	0.671	0.124	2.750	n.s.	n.s.	/
Campylobacterales	0.056	0.000	0.636	0.000	0.000	0.000	0.000	0.000	0.003	0.040	0.000	0.335	*	n.s.	/
Flavobacteriales	0.047	0.000	0.409	1.000	0.008	5.000	0.007	0.000	0.040	0.000	0.000	0.415	n.s.	n.s.	/
Sphingomonadales	0.009	0.000	0.486	0.005	0.001	0.464	0.000	0.000	0.000	0.000	0.000	0.047	**	*	-2.56
Rhizobiales	0.001	0.000	0.194	0.357	0.001	1.000	0.001	0.000	0.082	0.000	0.000	0.003	n.s.	n.s.	/
Acetobacterales	0.001	0.000	0.004	0.000	0.000	0.001	0.071	0.001	0.850	0.002	0.000	0.944	n.s.	n.s.	/
Enterobacteriales	0.000	0.000	0.002	0.001	0.000	0.534	0.000	0.000	0.012	0.000	0.000	0.135	n.s.	n.s.	/

Table S4. Relative abundance values and statistical analyses of the main bacteria families colonizing the gastric antrum (above) and corpus (below) in descending order of abundance in each group. Data are expressed as the median, first quartile (Q1) and third quartile (Q3). In the tables are reported the families with a percentage higher than 0.001%. KW represents the *p values* resulting from the Kruskal-Wallis test. Cuzick test for trend is expressed with the *p value* and, if significant, with the z value of trend. *p values* are annotated as *p<0.05,** p<0.01. n.s. represent a *p value* non-significant (p> 0.05). CTRL are patients with any gastric disease; mAG: patients with moderate atrophic gastritis (i.e. OLGA stage I-II); sAG: patients with severe atrophic gastritis (i.e. OLGA stage III-IV); HGD/GC: patients with severe atrophic gastritis and high-grade dysplasia or GC.

ANTRUM	CTRL			mAG			sAG			HGD/GC			KW	CUZICK	
	Median	Q1	Q3	Median	Q1	Q3	Median	Q1	Q3	Median	Q1	Q3	*p*	*p*	z
Streptococcaceae	29.000	21.000	37.000	36.000	31.000	52.000	46.500	30.250	50.250	35.000	26.750	58.750	n.s.	n.s.	/
Pasteurellaceae	10.000	2.000	12.500	2.000	0.257	8.000	5.000	0.532	13.000	3.000	1.186	11.500	n.s.	n.s.	/
Neisseriaceae	9.000	0.396	18.000	2.000	0.766	8.000	1.000	0.019	5.000	3.000	0.775	7.500	n.s.	n.s.	/
Micrococcaceae	5.000	0.970	7.500	3.000	0.627	6.000	5.000	3.000	10.500	6.000	4.000	9.750	n.s.	n.s.	/
Veillonellaceae	5.000	1.500	13.000	2.000	0.832	7.000	4.000	3.000	8.000	3.000	1.250	5.500	n.s.	n.s.	/
Family XI-01	3.000	2.000	8.500	5.000	2.000	9.000	3.500	1.250	6.000	4.000	1.500	7.000	n.s.	n.s.	/
Actinomycetaceae	2.000	2.000	3.000	1.000	0.635	3.000	1.500	0.706	3.000	0.911	0.516	1.000	n.s.	*	-2.25
Carnobacteriaceae	2.000	1.000	4.000	1.000	0.768	2.000	1.000	0.150	2.000	0.969	0.712	1.000	n.s.	*	-2.28
Leptotrichiaceae	2.000	0.230	2.000	0.313	0.007	0.637	0.004	0.001	0.390	0.592	0.034	1.000	n.s.	n.s.	/
Erysipelotrichaceae	1.000	0.509	2.000	0.490	0.007	2.000	0.575	0.007	1.000	0.545	0.008	0.966	n.s.	n.s.	/
Fusobacteriaceae	1.000	0.349	2.000	0.005	0.000	0.325	0.504	0.000	1.750	0.199	0.004	0.938	n.s.	n.s.	/
Lachnospiraceae	1.000	0.840	2.000	0.533	0.280	2.000	0.758	0.064	0.994	0.856	0.715	1.750	n.s.	n.s.	/
Saccharimonadaceae	1.000	1.000	2.500	1.000	0.013	4.000	0.533	0.002	2.000	0.381	0.007	0.987	n.s.	n.s.	/
Family XI-02	0.928	0.625	3.000	0.427	0.013	2.000	0.295	0.003	1.691	0.498	0.119	0.757	n.s.	n.s.	/
Prevotellaceae	0.812	0.044	2.500	0.001	0.000	0.125	0.050	0.001	0.610	0.105	0.004	0.609	n.s.	n.s.	/
Atopobiaceae	0.807	0.300	2.000	0.004	0.002	2.000	0.311	0.001	0.650	0.002	0.000	0.602	n.s.	**	-2.71
Family XIII	0.690	0.343	0.908	0.392	0.009	1.000	0.141	0.001	0.680	0.454	0.012	1.000	n.s.	n.s.	/
Peptostreptococcaceae	0.396	0.073	1.000	0.007	0.000	0.630	0.000	0.000	0.001	0.002	0.000	0.291	**	*	-2.41
Campylobacteraceae	0.370	0.001	0.617	0.000	0.000	0.001	0.000	0.000	0.123	0.000	0.000	0.047	n.s.	*	-2.09
Porphyromonadaceae	0.245	0.026	1.384	1.000	0.075	2.000	0.088	0.001	0.878	0.495	0.038	0.852	n.s.	n.s.	/
Propionibacteriaceae	0.148	0.070	0.253	0.060	0.001	0.255	0.296	0.053	0.381	0.143	0.055	0.519	n.s.	n.s.	/
Sphingomonadaceae	0.146	0.001	0.430	0.000	0.000	0.000	0.000	0.000	0.001	0.005	0.000	0.429	**	n.s.	/

Staphylococcaceae	0.145	0.005	0.474	0.001	0.000	0.145	0.020	0.000	0.251	0.000	0.000	0.055	n.s.	*	-2.09
Corynebacteriaceae	0.093	0.001	0.437	0.010	0.001	0.389	0.000	0.000	0.020	0.000	0.000	0.156	*	**	-2.68
Burkholderiaceae	0.048	0.010	0.134	0.528	0.101	0.903	0.480	0.212	0.633	0.131	0.022	0.175	**	n.s.	/
Pseudomonadaceae	0.039	0.000	0.091	0.002	0.001	0.394	0.000	0.000	0.136	0.001	0.000	0.051	*	n.s.	/
Lactobacillaceae	0.019	0.000	0.225	0.001	0.000	0.629	0.018	0.000	0.288	0.001	0.000	0.123	n.s.	n.s.	/
Flavobacteriaceae	0.010	0.000	0.075	0.000	0.000	0.001	0.000	0.000	0.000	0.000	0.000	0.040	n.s.	n.s.	/
Bifidobacteriaceae	0.006	0.000	0.116	0.000	0.000	0.002	0.000	0.000	0.142	0.000	0.000	0.004	n.s.	n.s.	/
Ruminococcaceae	0.004	0.001	0.315	0.001	0.000	0.002	0.002	0.000	0.072	0.004	0.000	0.107	n.s.	n.s.	/
Enterobacteriaceae	0.001	0.000	0.019	0.000	0.000	0.000	0.023	0.000	0.363	0.000	0.000	0.001	*	n.s.	/
Acetobacteraceae	0.001	0.000	0.260	0.000	0.000	0.002	0.026	0.000	0.462	0.141	0.003	0.245	n.s.	n.s.	/
Bacillaceae	0.001	0.000	0.016	0.000	0.000	0.000	0.000	0.000	0.001	0.008	0.000	0.336	**	n.s.	/
Weeksellaceae	0.001	0.000	0.046	0.003	0.001	0.037	0.000	0.000	0.002	0.000	0.000	0.572	n.s.	n.s.	/
Moraxellaceae	0.001	0.000	0.169	0.001	0.000	0.001	0.000	0.000	0.039	0.002	0.000	0.119	n.s.	n.s.	/
Enterococcaceae	0.000	0.000	0.002	0.009	0.000	0.176	0.019	0.001	0.179	0.002	0.000	0.543	n.s.	*	2.24

CORPUS	CTRL			mAG			sAG			HGD/GC			KW	CUZICK	
	Median	Q1	Q3	Median	Q1	Q3	Median	Q1	Q3	Median	Q1	Q3	*p*	*p*	z
Streptococcaceae	27.000	16.000	31.000	38.000	22.000	46.000	35.500	25.000	55.500	28.500	19.000	46.000	n.s.	n.s.	/
Family XI-01	4.000	2.000	7.500	4.000	2.000	12.000	3.000	2.000	4.750	2.000	1.000	4.750	n.s.	n.s.	/
Pasteurellaceae	4.000	2.000	7.500	2.000	0.010	3.000	3.000	1.000	8.750	8.000	0.710	12.000	n.s.	n.s.	/
Veillonellaceae	4.000	2.000	19.000	8.000	3.000	10.000	6.000	2.000	11.750	2.000	1.000	6.500	n.s.	*	-2.04
Actinomycetaceae	3.000	2.500	6.000	2.000	0.607	2.000	0.810	0.574	3.000	0.593	0.240	2.000	*	*	-2.54
Micrococcaceae	3.000	1.000	4.000	2.000	0.011	6.000	5.000	3.000	8.500	4.500	2.000	11.750	n.s.	n.s.	/
Carnobacteriaceae	2.000	1.000	4.500	0.454	0.002	0.958	1.000	0.568	2.000	1.000	0.678	1.000	n.s.	n.s.	/
Family XI-02	2.000	0.836	2.500	0.002	0.001	0.729	0.001	0.000	0.392	0.264	0.007	0.440	**	*	-2.55
Atopobiaceae	1.000	0.208	1.000	0.005	0.000	0.228	0.005	0.000	0.732	0.001	0.000	0.557	n.s.	*	-2.02
Erysipelotrichaceae	1.000	0.645	3.000	0.044	0.001	1.000	0.329	0.001	2.000	0.410	0.001	1.000	n.s.	n.s.	/
Family XIII	1.000	0.364	2.000	0.007	0.000	0.465	0.061	0.000	0.533	0.184	0.003	0.420	*	n.s.	/
Prevotellaceae	1.000	0.101	5.500	0.750	0.004	2.000	0.248	0.003	0.920	0.211	0.000	1.000	n.s.	n.s.	/

Saccharimonadaceae	1.000	0.999	3.500	0.097	0.005	1.000	0.056	0.004	2.000	0.156	0.002	0.579	n.s.	*	-2.16
Lachnospiraceae	0.969	0.221	1.500	0.006	0.002	1.000	0.211	0.002	0.912	0.514	0.002	1.000	n.s.	n.s.	/
Neisseriaceae	0.939	0.564	10.500	1.000	0.514	4.000	0.469	0.004	5.750	2.500	1.250	7.500	n.s.	n.s.	/
Corynebacteriaceae	0.573	0.151	2.000	0.731	0.002	1.000	0.029	0.000	0.330	0.001	0.000	0.691	n.s.	*	-2.51
Peptostreptococcaceae	0.515	0.022	1.500	0.001	0.000	0.010	0.000	0.000	0.001	0.002	0.000	0.358	**	*	-2.32
Bifidobacteriaceae	0.490	0.005	1.500	0.001	0.000	0.002	0.009	0.000	0.128	0.000	0.000	0.000	*	**	-2.87
Propionibacteriaceae	0.462	0.196	2.000	0.878	0.360	3.000	0.460	0.075	0.865	0.182	0.073	1.750	n.s.	n.s.	/
Staphylococcaceae	0.245	0.043	1.357	0.097	0.001	3.000	0.000	0.000	0.001	0.150	0.001	0.923	*	n.s.	/
Porphyromonadaceae	0.077	0.001	0.940	0.003	0.002	0.955	0.002	0.000	0.189	0.040	0.002	0.890	n.s.	n.s.	/
Campylobacteraceae	0.056	0.000	0.636	0.000	0.000	0.000	0.000	0.000	0.000	0.040	0.000	0.335	**	n.s.	/
Moraxellaceae	0.047	0.000	0.277	0.008	0.001	0.869	0.000	0.000	0.008	0.000	0.000	0.122	n.s.	n.s.	/
Lactobacillaceae	0.039	0.000	0.270	0.001	0.000	0.004	0.000	0.000	0.177	0.001	0.000	0.100	n.s.	n.s.	/
Fusobacteriaceae	0.038	0.002	1.500	0.001	0.000	0.434	0.007	0.001	0.131	0.170	0.000	0.747	n.s.	n.s.	/
Leptotrichiaceae	0.038	0.002	1.459	0.004	0.002	0.661	0.118	0.000	1.626	0.528	0.004	1.750	n.s.	n.s.	/
Sphingomonadaceae	0.009	0.000	0.486	0.005	0.001	0.464	0.000	0.000	0.000	0.000	0.000	0.047	**	*	-2.56
Burkholderiaceae	0.003	0.001	0.078	0.634	0.011	2.000	0.648	0.356	1.000	0.191	0.084	0.431	*	n.s.	/
Ruminococcaceae	0.002	0.000	0.310	0.001	0.000	0.002	0.001	0.000	0.007	0.000	0.000	0.002	n.s.	n.s.	/
Aerococcaceae	0.002	0.000	0.231	0.001	0.000	0.002	0.000	0.000	0.000	0.000	0.000	0.001	*	*	-2.36
Xanthobacteraceae	0.001	0.000	0.127	0.002	0.000	0.111	0.000	0.000	0.001	0.000	0.000	0.000	n.s.	n.s.	/
Acetobacteraceae	0.001	0.000	0.004	0.000	0.000	0.001	0.071	0.001	0.850	0.002	0.000	0.944	n.s.	n.s.	/
Weeksellaceae	0.000	0.000	0.382	0.757	0.005	5.000	0.005	0.000	0.040	0.000	0.000	0.000	*	*	-1.98
Bacillaceae	0.000	0.000	0.017	0.002	0.000	1.000	0.000	0.000	0.161	0.009	0.000	0.201	n.s.	n.s.	/
Enterobacteriaceae	0.000	0.000	0.002	0.001	0.000	0.534	0.000	0.000	0.012	0.000	0.000	0.135	n.s.	n.s.	/
Enterococcaceae	0.000	0.000	0.000	0.000	0.000	0.030	0.379	0.012	2.000	0.001	0.000	0.076	**	*	2.05

Table S5. Relative abundance values and statistical analyses of the main bacteria genera colonizing the gastric antrum (above) and corpus (below) in descending order of abundance in each group. Data are expressed as the median, first quartile (Q1) and third quartile (Q3). In the tables are reported the genera with a percentage higher than 0.001%. KW represents the *p values* resulting from the Kruskal-Wallis test. Cuzick test for trend is expressed with the *p value* and, if significant, with the z value of trend. *p values* are annotated as *$p<0.05$,** $p<0.01$. n.s. represent a *p value* non-significant ($p>0.05$). CTRL are patients with any gastric disease; mAG: patients with moderate atrophic gastritis (i.e. OLGA stage I-II); sAG: patients with severe atrophic gastritis (i.e. OLGA stage III-IV); HGD/GC: patients with severe atrophic gastritis and high-grade dysplasia or GC.

ANTRUM	CTRL			mAG			sAG			HGD/GC			KW	CUZICK	
	Median	Q1	Q3	Median	Q1	Q3	Median	Q1	Q3	Median	Q1	Q3	*p*	*p*	z
Streptococcus	29.000	21.000	37.000	36.000	31.000	52.000	46.500	30.250	50.250	35.000	26.750	58.750	n.s.	n.s.	/
Neisseria	9.000	0.396	18.000	2.000	0.686	8.000	1.000	0.019	5.000	3.000	0.775	7.500	n.s.	n.s.	/
Haemophilus	8.000	1.000	11.500	2.000	0.256	7.000	4.000	0.280	11.750	3.000	1.186	10.750	n.s.	n.s.	/
Rothia	5.000	0.970	7.500	3.000	0.361	6.000	5.000	3.000	10.500	6.000	3.250	9.750	n.s.	n.s.	/
Gemella	3.000	2.000	8.500	5.000	2.000	9.000	3.500	1.250	6.000	4.000	1.500	7.000	n.s.	n.s.	/
Veillonella	3.000	0.927	12.000	2.000	0.832	7.000	3.000	2.250	7.750	3.000	1.250	5.250	n.s.	n.s.	/
Actinomyces	2.000	2.000	3.000	1.000	0.635	3.000	1.500	0.705	3.000	0.911	0.516	1.000	n.s.	*	-2.25
Granulicatella	2.000	1.000	4.000	1.000	0.618	2.000	1.000	0.150	2.000	0.969	0.710	1.000	n.s.	*	-2.15
Leptotrichia	2.000	0.230	2.000	0.313	0.007	0.637	0.004	0.001	0.390	0.592	0.034	1.000	n.s.	n.s.	/
Fusobacterium	1.000	0.349	2.000	0.005	0.000	0.325	0.504	0.000	1.750	0.199	0.004	0.938	n.s.	n.s.	/
TM7 phylum sp.	1.000	0.393	2.500	1.000	0.007	3.000	0.533	0.002	2.000	0.381	0.002	0.982	n.s.	n.s.	/
Parvimonas	0.928	0.362	3.000	0.427	0.013	2.000	0.118	0.002	1.608	0.413	0.022	0.745	n.s.	n.s.	/
Prevotella	0.813	0.043	2.476	0.001	0.000	0.125	0.050	0.001	0.607	0.043	0.002	0.598	*	n.s.	/
Atopobium	0.807	0.298	2.000	0.004	0.002	2.000	0.311	0.001	0.650	0.002	0.000	0.602	n.s.	**	-2.73
Megasphaera	0.676	0.103	1.500	0.001	0.000	0.374	0.157	0.000	0.600	0.130	0.004	0.282	n.s.	n.s.	/
Peptostreptococcus	0.396	0.071	1.000	0.007	0.000	0.485	0.000	0.000	0.001	0.001	0.000	0.291	**	**	-2.63
Campylobacter	0.370	0.001	0.617	0.000	0.000	0.001	0.000	0.000	0.123	0.000	0.000	0.047	n.s.	*	-2.3
Porphyromonas	0.245	0.026	1.384	1.000	0.075	2.000	0.088	0.001	0.878	0.495	0.038	0.852	n.s.	n.s.	/
Oribacterium	0.224	0.008	0.711	0.001	0.000	0.607	0.141	0.001	0.766	0.696	0.285	0.896	n.s.	n.s.	/
Staphylococcus	0.145	0.005	0.474	0.001	0.000	0.145	0.000	0.000	0.063	0.000	0.000	0.055	n.s.	*	-2.51
Mogibacterium	0.120	0.029	0.296	0.000	0.000	0.000	0.000	0.000	0.096	0.047	0.004	0.329	n.s.	n.s.	/
Corynebacterium	0.093	0.000	0.319	0.010	0.001	0.389	0.000	0.000	0.001	0.000	0.000	0.022	**	*	-2.18

Cutibacterium	0.089	0.040	0.214	0.060	0.001	0.255	0.296	0.053	0.381	0.143	0.034	0.519	n.s.	n.s.	/
Pseudomonas	0.039	0.000	0.091	0.002	0.001	0.394	0.000	0.000	0.136	0.001	0.000	0.051	n.s.	n.s.	/
Solobacterium	0.036	0.000	0.243	0.000	0.000	0.049	0.000	0.000	0.002	0.000	0.000	0.018	n.s.	n.s.	/
Lactobacillus	0.019	0.000	0.225	0.001	0.000	0.629	0.018	0.000	0.288	0.001	0.000	0.123	n.s.	n.s.	/
Aggregatibacter	0.004	0.000	1.129	0.000	0.000	0.004	0.001	0.000	0.374	0.000	0.000	0.001	n.s.	*	-2.01
Stomatobaculum	0.001	0.000	0.031	0.000	0.000	0.002	0.000	0.000	0.334	0.001	0.000	0.355	n.s.	n.s.	/
Actinobacillus	0.001	0.000	0.174	0.000	0.000	0.017	0.001	0.000	0.168	0.000	0.000	0.128	n.s.	n.s.	/
Acetobacter	0.001	0.000	0.260	0.000	0.000	0.002	0.026	0.000	0.462	0.057	0.000	0.178	n.s.	n.s.	/
Bacillus	0.001	0.000	0.007	0.000	0.000	0.000	0.000	0.000	0.000	0.003	0.000	0.117	**	n.s.	/
Acinetobacter	0.000	0.000	0.158	0.000	0.000	0.001	0.000	0.000	0.039	0.002	0.000	0.119	n.s.	n.s.	/
Enterococcus	0.000	0.000	0.002	0.009	0.000	0.176	0.019	0.001	0.179	0.002	0.000	0.543	n.s.	*	2.13
Mycobacterium	0.000	0.000	0.013	0.000	0.000	0.000	0.000	0.000	0.061	0.004	0.000	0.015	n.s.	n.s.	/
Paracoccus	0.000	0.000	0.009	0.000	0.000	0.034	0.000	0.000	0.000	0.002	0.000	0.174	n.s.	n.s.	/
Pelomonas	0.000	0.000	0.000	0.025	0.000	0.097	0.124	0.002	0.581	0.037	0.000	0.148	*	*	2.05

CORPUS	CTRL			mAG			sAG			HGD/GC			KW	CUZICK	
	Median	Q1	Q3	Median	Q1	Q3	Median	Q1	Q3	Median	Q1	Q3	*p*	*p*	z
Streptococcus	25.000	15.500	31.500	31.000	22.000	46.000	35.500	25.000	52.750	28.500	19.000	46.000	n.s.	n.s.	/
Haemophilus	5.000	2.250	6.750	0.379	0.010	3.000	3.000	1.500	9.750	7.000	0.843	11.750	n.s.	n.s.	/
Gemella	3.500	2.000	8.000	6.000	2.000	12.000	3.000	2.250	4.750	2.000	0.569	4.750	n.s.	n.s.	/
Veillonella	3.000	2.000	13.250	6.000	3.000	10.000	7.500	2.000	11.750	2.000	1.000	5.500	n.s.	n.s.	/
Actinomyces	2.500	1.250	3.000	2.000	0.406	2.000	1.437	0.592	3.000	0.593	0.240	2.000	n.s.	*	-2.06
Rothia	2.500	1.000	3.000	2.000	0.011	5.000	4.500	2.250	8.500	5.500	2.250	17.500	n.s.	*	1.99
Prevotella	2.403	0.311	5.175	0.014	0.002	0.936	0.248	0.002	0.920	0.211	0.000	1.268	n.s.	n.s.	/
Granulicatella	2.000	1.000	4.750	0.454	0.002	2.000	1.000	0.574	2.000	1.000	0.678	1.000	n.s.	n.s.	/
Neisseria	1.970	0.632	12.250	0.522	0.010	4.000	0.915	0.004	5.750	2.500	1.250	7.500	n.s.	n.s.	/
Parvimonas	1.500	0.365	2.000	0.003	0.002	0.729	0.001	0.000	0.320	0.070	0.004	0.402	**	*	-2.39
TM7 phylum sp.	0.923	0.587	1.750	0.941	0.002	3.000	0.011	0.001	2.000	0.070	0.000	0.476	n.s.	*	-2.33
Atopobium	0.768	0.193	1.000	0.005	0.000	0.575	0.004	0.000	0.732	0.012	0.000	0.547	n.s.	*	-2.2

Cutibacterium	0.580	0.199	2.500	0.440	0.273	3.000	0.478	0.170	0.764	0.106	0.025	1.750	n.s.	n.s.	/
Peptostreptococcus	0.540	0.013	1.732	0.001	0.000	0.122	0.000	0.000	0.001	0.000	0.000	0.358	**	**	-2.73
Staphylococcus	0.441	0.022	1.678	0.097	0.001	1.000	0.000	0.000	0.001	0.002	0.001	0.768	**	n.s.	/
Oribacterium	0.236	0.021	0.846	0.002	0.000	0.867	0.117	0.001	0.447	0.000	0.000	0.417	n.s.	n.s.	/
Leptotrichia	0.230	0.002	1.730	0.002	0.001	0.004	0.322	0.000	2.000	0.528	0.002	1.750	n.s.	n.s.	/
Megasphaera	0.229	0.024	2.343	0.520	0.001	0.902	0.099	0.000	0.238	0.022	0.001	0.065	n.s.	*	-1.99
Fusobacterium	0.200	0.006	1.750	0.001	0.000	0.434	0.002	0.000	0.131	0.174	0.000	0.747	n.s.	n.s.	/
Porphyromonas	0.173	0.002	0.970	0.003	0.001	0.955	0.002	0.000	0.189	0.040	0.002	0.890	**	n.s.	/
Corynebacterium	0.148	0.020	1.195	0.731	0.003	1.185	0.023	0.000	0.330	0.008	0.000	0.433	n.s.	n.s.	/
Lactobacillus	0.072	0.000	0.295	0.001	0.000	0.004	0.000	0.000	0.165	0.002	0.000	0.112	n.s.	n.s.	/
Mogibacterium	0.035	0.000	0.218	0.000	0.000	0.000	0.001	0.000	0.228	0.000	0.000	0.104	n.s.	n.s.	/
Solobacterium	0.009	0.002	0.142	0.000	0.000	0.000	0.000	0.000	0.003	0.001	0.000	0.002	**	n.s.	/
Acetobacter	0.001	0.000	0.005	0.000	0.000	0.001	0.024	0.000	0.397	0.003	0.000	0.670	n.s.	n.s.	/
Enterococcus	0.000	0.000	0.000	0.000	0.000	0.030	0.379	0.013	2.000	0.001	0.000	0.024	**	n.s.	/

Table S6. PERMANOVA analysis results according to different beta-diversity indices. Comparison of beta-diversity between non-dysplastic AG and dysplastic/cancer AG patients in both antrum and corpus microbial composition.

	ANTRUM		CORPUS	
	F statistic	*p value*	F statistic	*p value*
Bray-Curtis	2.63	0.001	3.78	<0.0001
Jaccard	1.98	<0.001	2.55	<0.0001
Unweighted Unifrac	2.18	0.042	5.03	<0.0001
Weighted Unifrac	0.61	0.785	1.45	<0.0001

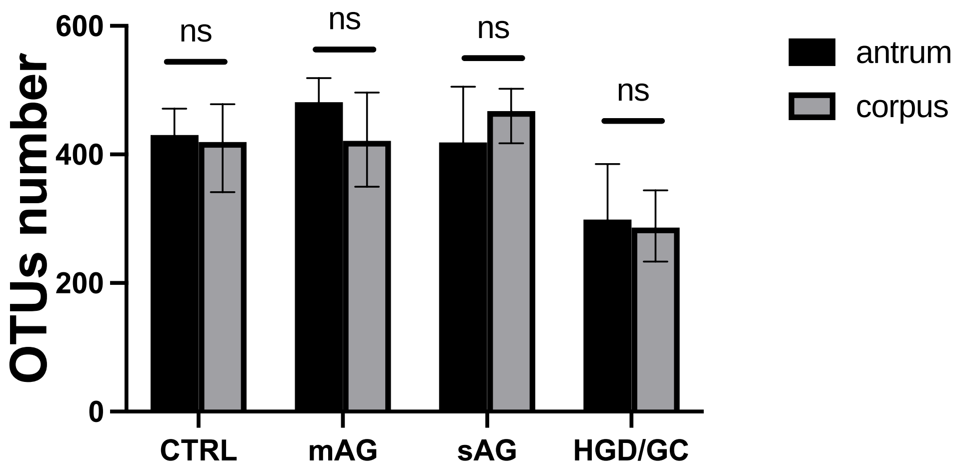

Figure S1. Comparison of the number of OTUs between antrum and corpus at each stage of the disease. Box plots represent the median and interquartile range. Post hoc analyses were annotated as ns=not significant.
